# Temporal Patterns of Body Composition After Heart and Lung Transplantation Assessed by Bioelectrical Impedance Analysis

**DOI:** 10.3390/jcm15187034

**Published:** 2026-09-11

**Authors:** Michał Bohdan, Anna Kowalczys, Alicja Radtke-Łysek, Wioletta Raczyńska, Alicja Fedyczkowska, Aleksandra Gutowska, Anna Borzyszkowska, Sławomir Żegleń, Marcin Gruchała

**Affiliations:** 1 First Department of Cardiology, Medical University of Gdańsk, Smoluchowskiego 17 Str., 80-214 Gdansk, Poland; 2 University Clinical Centre, Smoluchowskiego 17 Str., 80-952 Gdansk, Poland; 3 Division of Pulmonology, Medical University of Gdańsk, Smoluchowskiego 17 Str., 80-214 Gdansk, Poland

**Keywords:** thoracic organ transplantation, heart transplantation, lung transplantation, bioelectrical impedance analysis, body composition, skeletal muscle mass, sarcopenic obesity, phase angle, rehabilitation, nutritional assessment

## Abstract

**Background/Objectives:** Body composition may influence functional capacity and long-term outcomes after thoracic organ transplantation. However, direct comparisons of body composition patterns between heart transplant (HTx) and lung transplant (LTx) recipients remain limited. This study compared body composition parameters and their relationship with time since transplantation in HTx and LTx recipients using bioelectrical impedance analysis (BIA). **Methods:** This cross-sectional study included 79 clinically stable transplant recipients (38 HTx and 41 LTx) with a median time of 9 months after transplantation (range, 1 month–9 years). Body composition was assessed using the SECA mBCA 515 analyzer. Absolute fat mass (AFM), relative fat mass (RFM), fat-free mass (FFM), skeletal muscle mass (SMM), phase angle (PA), and extracellular water-to-total body water ratio (ECW/TBW) were measured. Associations between body composition parameters and time since transplantation were modelled using adjusted generalized additive models with separate smooth functions for HTx and LTx recipients. **Results:** Positive cross-sectional associations between AFM and time since transplantation were observed during the 3–9-month post-transplant interval in both groups (HTx: 0.72 kg/month, *p* = 0.042; LTx: 0.83 kg/month, *p* = 0.021). In contrast, significant positive associations between SMM and time since transplantation were observed exclusively among LTx recipients during the 1–3-month (0.59 kg/month, *p* = 0.039) and 3–9-month (0.49 kg/month, *p* = 0.003) intervals, whereas no significant associations were observed after HTx. In LTx recipients, ECW/TBW was negatively associated and PA positively associated with time since transplantation during the 3–9-month interval. **Conclusions:** HTx and LTx recipients exhibited different cross-sectional patterns of body composition according to time since transplantation. HTx recipients were characterized by greater adiposity without a significant association between SMM and time since transplantation, whereas LTx recipients showed positive associations between SMM and time since transplantation during the early post-transplant intervals.

## 1. Introduction

Heart transplantation (HTx) remains the gold standard for patients with advanced heart failure (HF), offering improved survival rates and quality of life among carefully selected individuals [1]. Similarly, lung transplantation (LTx) is considered for adults at high risk of death from lung disease within two years, provided there are no contraindications and a high probability of five-year post-transplant survival is anticipated [2]. Both HTx and LTx recipients require lifelong immunosuppressive therapy to prevent graft rejection. Although highly effective, immunosuppressive treatment is associated with metabolic complications, changes in body composition, weight gain, muscle loss, and an increased cardiovascular risk [3,4]. Consequently, obesity and adverse body composition changes are frequently observed after transplantation and contribute to increased cardiovascular risk [5,6]. In addition, data suggest that obese HTx recipients have a significantly higher risk of death, primary graft dysfunction, and any treated organ rejection [7].

Beyond obesity alone, increasing attention has been paid to sarcopenic obesity, characterized by excessive fat accumulation accompanied by reduced skeletal muscle mass or function, which may adversely affect clinical outcomes after transplantation. Conventional anthropometric measures such as BMI do not allow for distinguishing between fat mass, skeletal muscle mass, and body water distribution. Bioelectrical impedance analysis (BIA) enables comprehensive assessment of these compartments and has therefore become a useful tool for nutritional and metabolic evaluation in transplant recipients [8,9,10]. Consequently, BIA is increasingly used in the clinical assessment of patients with cachexia, sarcopenia, HF, chronic lung disease, and transplant recipients. Given the lack of direct comparisons between HTx and LTx recipients, it remains unclear whether body composition differs according to time since transplantation in a similar manner in these two populations. The novelty of the present study lies in the direct comparison of HTx and LTx recipients across a broad range of post-transplant time points and in the use of generalized additive models to characterize potentially non-linear, time-related patterns in multiple BIA-derived body composition parameters within a cross-sectional framework.

The aim of this study was to compare body composition parameters between HTx and LTx recipients and to evaluate their cross-sectional associations with time since transplantation using BIA and adjusted non-linear regression models.

## 2. Materials and Methods

### 2.1. Study Population

**Figure 1:**
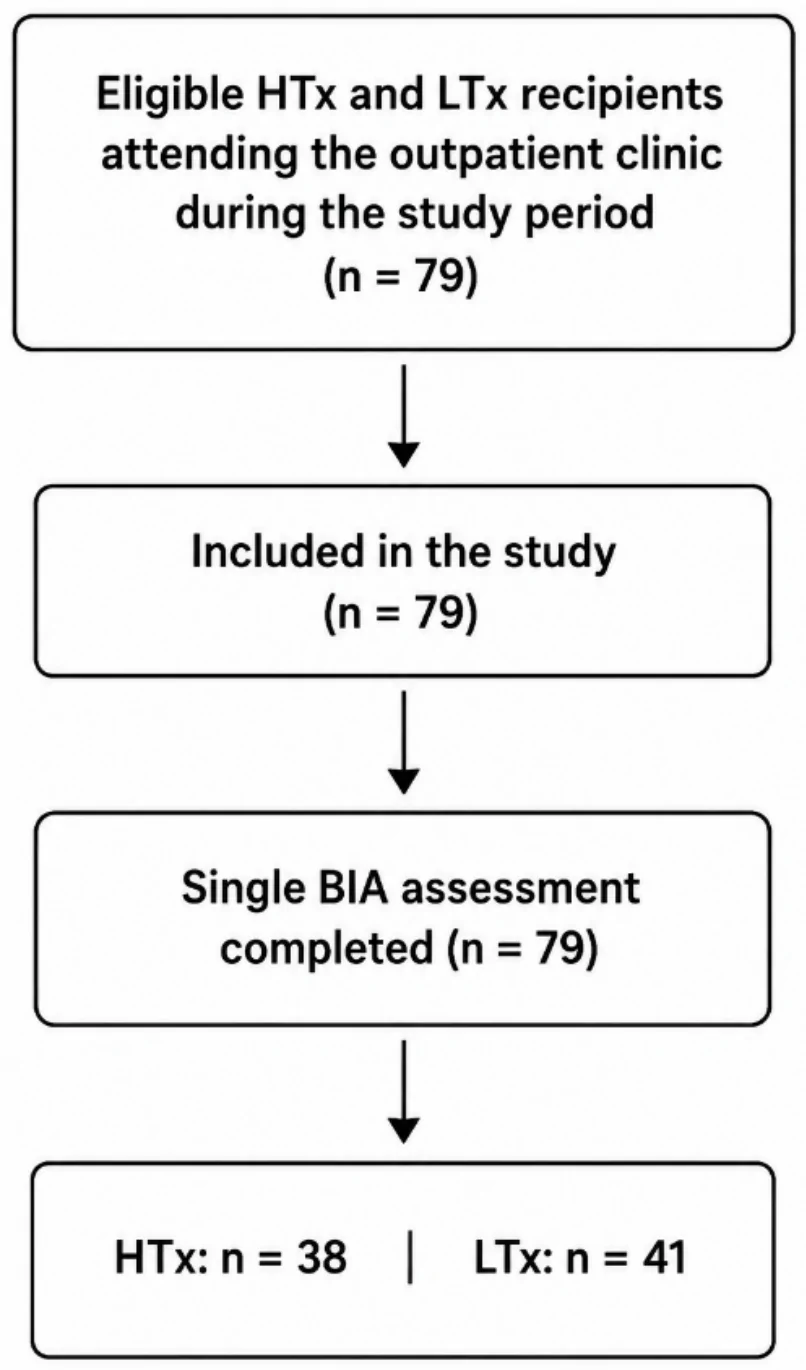
Flowchart of participant recruitment and study procedures. Eligible heart transplant (HTx) and lung transplant (LTx) recipients attending the outpatient clinic during the study period were included in the cross-sectional study and underwent a single bioelectrical impedance analysis (BIA) assessment. The final study population comprised 79 participants (38 HTx and 41 LTx recipients).

This cross-sectional observational study included 79 clinically stable transplant recipients, comprising 38 HTx and 41 LTx recipients. Participants were recruited among adult HTx and LTx recipients attending routine outpatient visits at the transplant clinic during the study period. All eligible clinically stable patients attending the clinic during this period were considered for inclusion. Each participant underwent a single study-specific assessment consisting of one BIA measurement performed during the outpatient visit. No repeated study measurements or prospective or retrospective longitudinal follow-up were performed as part of the study protocol. The time since transplantation was calculated as the interval between the date of transplantation and the date of the single BIA assessment. The median time since transplantation was 9 months (range: 1 month–9 years). Inclusion criteria were age ≥ 18 years and clinically stable heart or lung transplant status (Figure 1).

Body composition was assessed using the SECA mBCA 515 Medical Body Composition Analyzer (seca GmbH, Hamburg, Germany). The seca mBCA 515 is a multifrequency, eight-electrode bioelectrical impedance analyzer that performs segmental measurements using hand and foot electrodes. The device applies a measurement current of 100 μA across multiple frequencies ranging from 1 to 1000 kHz and measures impedance, resistance, reactance, and phase angle. The measurements were taken in the outpatient clinic in the morning during routine medical visits. Patients were asked to avoid eating or drinking for at least 4 h and avoid caffeine and alcohol intake for at least 12 h before the BIA. In addition, the patients were asked to refrain from vigorous exercise for 12 h before testing. The measurements were assessed in the standing position on a floor-based BIA device. The following parameters were evaluated: absolute fat mass (AFM, kg), relative fat mass (RFM, %), fat-free mass (FFM, kg), skeletal muscle mass (SMM, kg), extracellular water-to-total body water ratio (ECW/TBW, %), and phase angle (PA, °).

To facilitate the interpretation of cross-sectional associations with time since transplantation, participants were categorized into quartiles according to time since transplantation using common quartile cut-off values for both HTx and LTx recipients (Q1: 1–3 months, Q2: 3–9 months, Q3: 9–15 months, and Q4: 15–42.1 months). Associations between body composition parameters and time since transplantation were modelled using adjusted generalized additive models. Estimated average monthly cross-sectional slopes were subsequently calculated and presented for each quartile interval.

The study was conducted in accordance with the Declaration of Helsinki and approved by the Institutional Bioethics Committee (approval No. NKBBN/621/2020). Written informed consent was obtained from all participants prior to enrolment.

### 2.2. Statistical Analysis

Continuous variables are presented as mean ± standard deviation (SD) or median with interquartile range (IQR), as appropriate, whereas categorical variables are presented as counts and percentages. Baseline differences between HTx and LTx recipients were assessed using Student’s *t*-test or the Mann–Whitney U test for continuous variables and the χ^2^ test or Fisher’s exact test for categorical variables, as appropriate.

The relationships between body composition parameters and time since transplantation were modelled using generalized additive models to account for potential non-linear associations. Separate smooth functions of time since transplantation were estimated for HTx and LTx recipients, and transplant type was additionally included as a fixed effect. The models were adjusted for age, sex, and height. Time since transplantation was modelled using a penalized thin-plate regression spline (k = 5), whereas age and height were modelled using penalized thin-plate regression splines with low complexity (k = 3). Model parameters were estimated using restricted maximum likelihood (REML). Before model fitting, time since transplantation was winsorized at the 95th percentile to reduce the influence of extreme values while retaining all observations.

Estimated effects represent the average cross-sectional difference in the analyzed body composition parameter associated with a one-month difference in time since transplantation. For descriptive purposes and to facilitate interpretation of the results, estimated cross-sectional slopes, derived from the first derivative of the fitted smooth functions, were summarized within common quartile intervals of time since transplantation (Q1–Q4). Quartiles were used exclusively for presentation of the results and were not included as predictors in the statistical models. In addition, points at which the centered partial smooth crossed zero were identified, and average monthly cross-sectional slopes were estimated before and after these model-derived reference points.

Statistical analyses were performed using R version 4.5.0 (R Foundation for Statistical Computing, Vienna, Austria), including the mgcv and gratia packages. A two-sided *p* value < 0.05 was considered statistically significant.

## 3. Results

**Figure 2:**
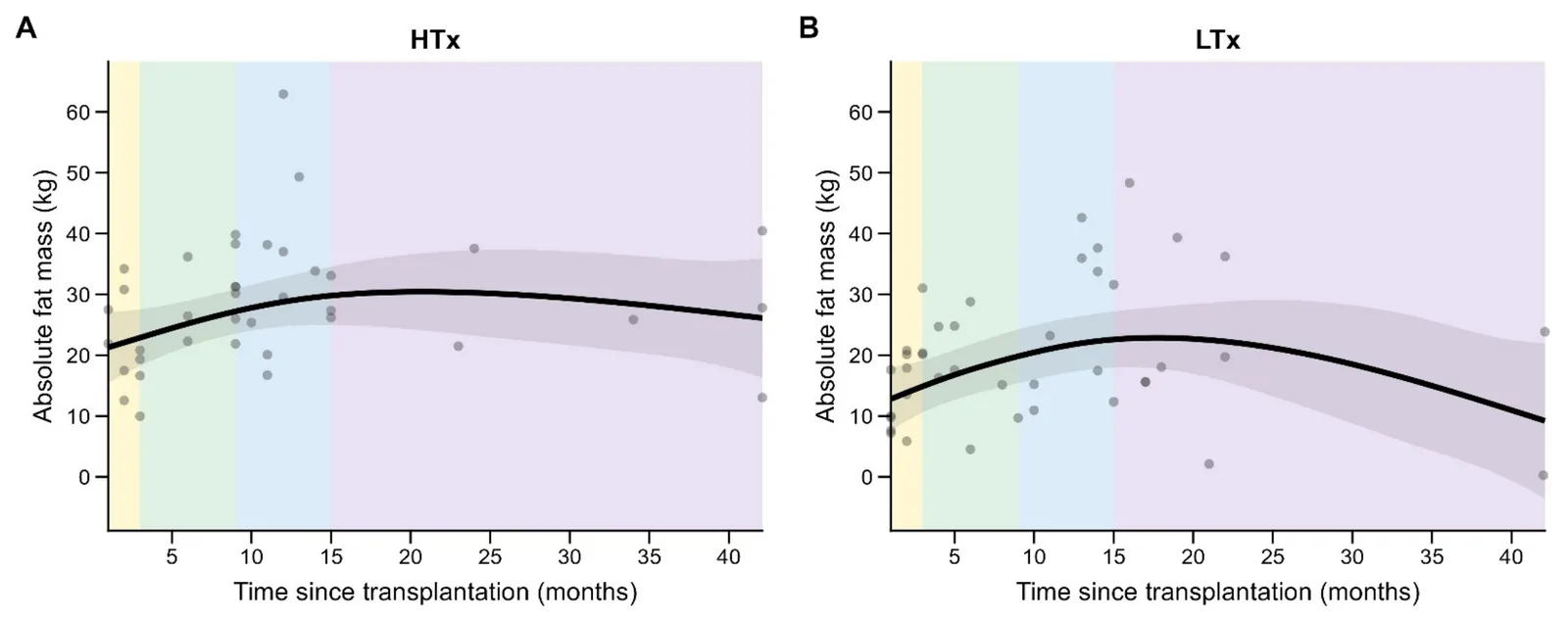
Absolute fat mass according to time since transplantation. Adjusted GAM-fitted values with 95% confidence intervals are shown separately for HTx (**A**) and LTx (**B**) recipients; points represent individual observations. The fitted curves represent cross-sectional associations with time since transplantation and should not be interpreted as within-person longitudinal trajectories.

**Figure 3:**
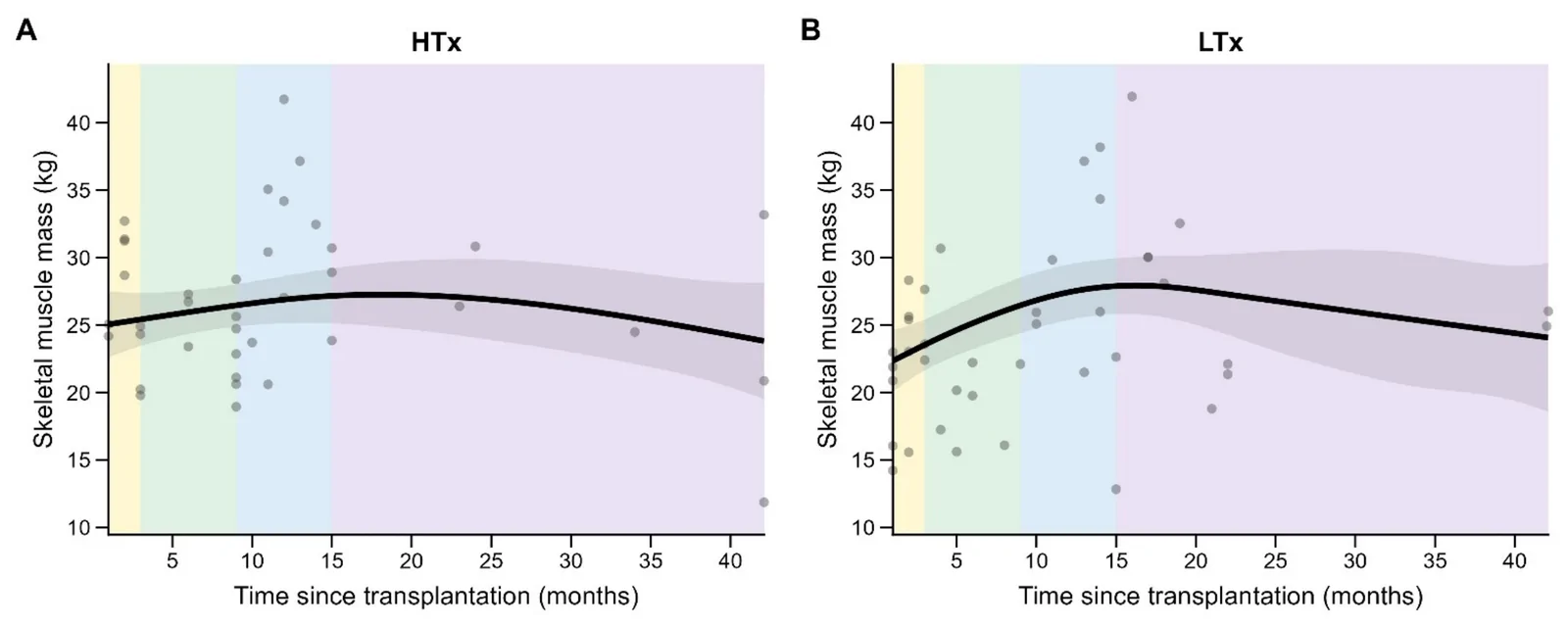
Skeletal muscle mass according to time since transplantation. Adjusted GAM-fitted values with 95% confidence intervals are shown separately for HTx (**A**) and LTx (**B**) recipients; points represent individual observations. The fitted curves represent cross-sectional associations with time since transplantation and should not be interpreted as within-person longitudinal trajectories.

**Figure 4:**
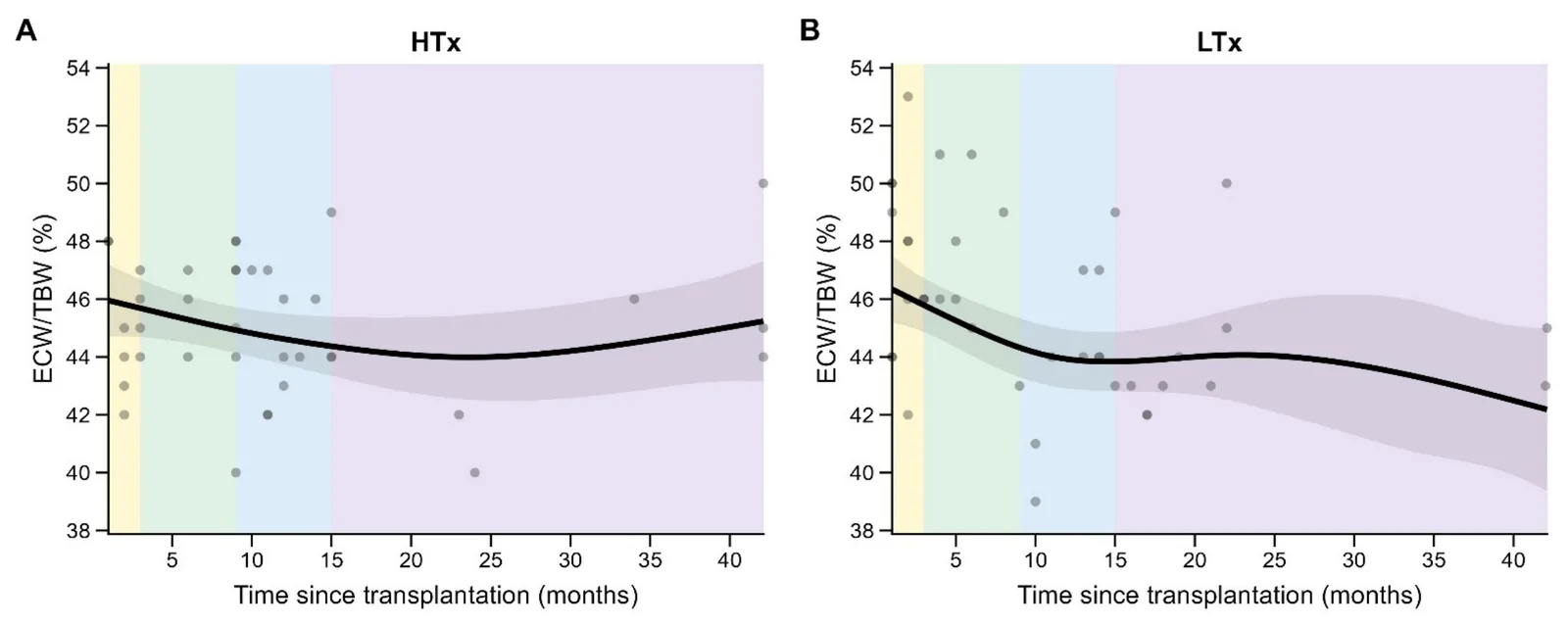
ECW/TBW according to time since transplantation. Adjusted GAM-fitted values with 95% confidence intervals are shown separately for HTx (**A**) and LTx (**B**) recipients; points represent individual observations. The fitted curves represent cross-sectional associations with time since transplantation and should not be interpreted as within-person longitudinal trajectories.

**Figure 5:**
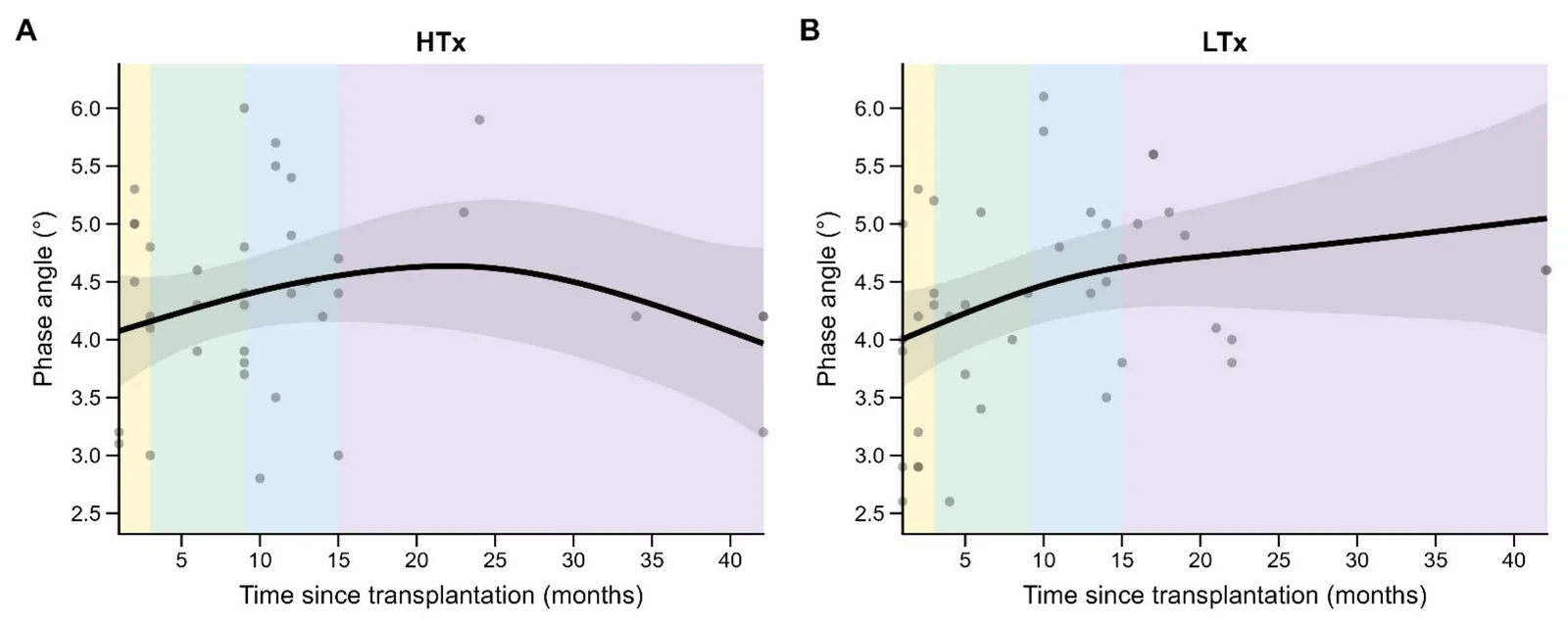
Phase angle according to time since transplantation. Adjusted GAM-fitted values with 95% confidence intervals are shown separately for HTx (**A**) and LTx (**B**) recipients; points represent individual observations. The fitted curves represent cross-sectional associations with time since transplantation and should not be interpreted as within-person longitudinal trajectories.

Baseline characteristics of the study population and body composition are presented in Table 1. HTx recipients exhibited significantly higher absolute and relative fat mass compared to LTx recipients. No statistically significant differences were observed between the groups regarding FFM, SMM, PA, or ECW/TBW ratio. AFM showed a significant positive cross-sectional association with time since transplantation during the second post-transplant interval (3–9 months) in both HTx (0.72 kg/month, *p* = 0.042) and LTx recipients (0.83 kg/month, *p* = 0.021) (Figure 2). Spline models indicated positive, although non-significant, cross-sectional slopes for RFM in both groups during the early post-transplant intervals. In LTx recipients, RFM showed a significant negative association with time since transplantation during the fourth interval (15–42.1 months; −0.48%/month, *p* = 0.021). No significant associations between SMM and time since transplantation were observed in HTx recipients, whereas significant positive associations were identified in LTx recipients during the first (0.59 kg/month, *p* = 0.039) and second (0.49 kg/month, *p* = 0.003) intervals (Figure 3). Similarly, FFM showed no significant associations with time since transplantation in HTx recipients, whereas a significant positive association was observed in LTx recipients during the second interval (0.52 kg/month, *p* = 0.033). ECW/TBW showed a significant negative association with time since transplantation in LTx recipients during the second interval (−0.24% per month, *p* = 0.004), whereas the corresponding associations in HTx recipients were not statistically significant (Figure 4). PA showed a significant positive association with time since transplantation in LTx recipients during the second interval (0.05°/month, *p* = 0.027) (Figure 5), whereas no statistically significant associations were observed in HTx recipients.

Detailed estimates of adjusted cross-sectional slopes are presented in Table 2.

## 4. Discussion

The main finding of the present study is that heart and lung transplant recipients exhibit distinct body composition profiles according to time since transplantation. HTx recipients had greater adiposity than LTx recipients and showed no significant association between SMM and time since transplantation, whereas LTx recipients showed significant positive associations of SMM and FFM with time since transplantation during the early post-transplant intervals. AFM was positively associated with time since transplantation during the 3–9-month interval in both groups. In addition, LTx recipients showed a negative association of ECW/TBW and a positive association of PA with time since transplantation during this interval. Taken together, these findings indicate distinct cross-sectional body composition patterns according to time since heart and lung transplantation.

Post-transplant weight gain and increased adiposity have been consistently reported after both HTx and LTx [11,12,13,14]. Previous studies suggest that this increase is frequently driven predominantly by fat accumulation rather than proportional restoration of lean tissue [14]. Our findings are consistent with these observations, as AFM showed a significant positive association with time since transplantation during the 3–9-month interval in both transplant groups. The particularly unfavorable profile observed after HTx, characterized by higher adiposity without a significant positive association between SMM and time since transplantation, may be clinically relevant because the combination of excess adiposity and relatively low muscle mass may contribute to a sarcopenic obesity phenotype. Hasse et al. similarly reported increasing fat mass during the first year after HTx, accompanied by reductions in FFM [15], while Ram et al. observed substantial post-HTx weight gain predominantly attributable to increased fat mass [16]. These findings support the concept of evaluating body composition rather than measuring body weight or BMI alone after thoracic organ transplantation.

A major difference between the two transplant groups concerned SMM. In our study, SMM showed no significant association with time since transplantation after HTx, whereas significant positive associations were observed in LTx recipients during the 1–3- and 3–9-month intervals, accompanied by a positive association of FFM during the latter interval. Previous studies have also documented impaired restoration of lean tissue after HTx. Braith et al. reported loss of lean body mass during the first six months after HTx in patients not participating in structured rehabilitation, whereas resistance training improved skeletal muscle strength and helped counteract steroid-associated myopathy [17]. Conversely, increases in lean mass have been reported after LTx. Oshima et al. observed increases in both fat mass and fat-free mass index, particularly during the first post-transplant year [18], which is consistent with the positive cross-sectional associations of SMM and FFM with time since transplantation observed in our LTx recipients.

The different muscle patterns after HTx and LTx may partly reflect differences in pre-transplant disease burden. Candidates for LTx frequently experience substantial muscle wasting associated with chronic respiratory insufficiency, hypoxemia, physical inactivity, and systemic catabolism. Restoration of respiratory function after transplantation, together with increased physical activity and rehabilitation, may therefore contribute to greater skeletal muscle mass at later post-transplant time points. In contrast, persistent skeletal muscle dysfunction and the metabolic effects of immunosuppressive therapy may contribute to the absence of a positive association between lean tissue parameters and time since transplantation after HTx. However, because pre-transplant body composition and detailed rehabilitation data were unavailable in our study, these mechanisms remain hypothetical and require confirmation in longitudinal studies.

The associations of ECW/TBW and PA with time since transplantation provide additional evidence of differences between the transplant groups. LTx recipients demonstrated a significant negative association between ECW/TBW and time since transplantation and a positive association between PA and time since transplantation during the 3–9-month interval, whereas the corresponding associations after HTx were not statistically significant. ECW/TBW reflects fluid distribution, while PA has been used as an indicator of cellular integrity and nutritional status. Previous studies have shown elevated ECW/TBW in patients with cardiovascular disease and volume overload [19,20], and PA has been investigated as a marker of nutritional and functional status in transplant recipients [15]. Thus, the lower ECW/TBW and higher PA values associated with later time points within this interval may be consistent with differences in fluid balance and cellular condition. However, these cross-sectional associations should not be interpreted as evidence of within-patient improvement over time.

From a clinical perspective, our findings support body composition assessment as a complementary tool to conventional weight and BMI monitoring after thoracic organ transplantation. BMI does not allow differentiation between adipose tissue, skeletal muscle, and body water, whereas BIA provides information on each of these compartments. The different profiles observed in HTx and LTx recipients suggest that post-transplant management may require different priorities. In HTx recipients, the combination of greater adiposity and the absence of a significant positive association between SMM and time since transplantation suggests particular attention to prevention of excessive fat accumulation and preservation or restoration of muscle mass through nutritional management and structured rehabilitation. In LTx recipients, the positive association between skeletal muscle mass and time since transplantation during the early post-transplant intervals suggests that continued monitoring of both adiposity and muscle mass may be warranted. Importantly, the present study was not designed to determine whether these body composition patterns influence graft function, cardiovascular events, or survival, and therefore their prognostic significance requires prospective evaluation.

### Study Limitations

Several limitations should be acknowledged. First, this was a single-center study with a relatively small and heterogeneous sample. Most importantly, the cross-sectional design precluded direct assessment of within-patient longitudinal changes in body composition. Each participant underwent a single assessment at a different time after transplantation; therefore, the observed patterns represent cross-sectional associations with time since transplantation rather than individual temporal trajectories. Although generalized additive models allowed us to characterize potentially non-linear associations with time since transplantation, they do not overcome this inherent limitation of the study design. Comparisons between individuals assessed at different post-transplant time points may also be affected by cohort effects, as these patients may differ in clinical characteristics, treatment exposure, pre-transplant condition, or other unmeasured factors. Survivorship bias should additionally be considered, particularly at later post-transplant time points, because patients available for assessment may represent a selected group of survivors with different clinical or body composition profiles from those who died or were otherwise unavailable for assessment. Consequently, the observed associations should not be interpreted as within-patient longitudinal changes or causal temporal trajectories.

In addition, several potentially important confounding factors were not incorporated into the statistical models. Detailed and standardized information on immunosuppressive regimens and cumulative corticosteroid exposure, rehabilitation participation, habitual physical activity, dietary intake and nutritional interventions was not systematically available. Pre-transplant body composition and nutritional status were also unavailable, preventing assessment of whether some of the observed differences between HTx and LTx recipients were already present before transplantation. These unmeasured factors may have influenced both fat and muscle mass and may partly explain the observed between-group differences. Therefore, residual confounding cannot be excluded.

Moreover, BIA has inherent methodological limitations, particularly in transplant recipients in whom fluid status may vary. Although measurements were performed under standardized conditions during morning outpatient visits, with restrictions on food and fluid intake, caffeine, alcohol, and vigorous exercise before testing, hydration status could not be fully standardized. In particular, information on the timing and dose of diuretic treatment was not systematically collected. Fluctuations in extracellular fluid volume, peripheral edema, and diuretic use may influence electrical impedance and consequently BIA-derived estimates, particularly FFM and SMM. BIA was selected because it is non-invasive, rapid, repeatable, and readily applicable in routine clinical practice rather than because it represents a reference method for body composition assessment. Accordingly, BIA-derived estimates should not be considered interchangeable with measurements obtained using reference imaging-based methods such as dual-energy X-ray absorptiometry or computed tomography.

Finally, owing to the modest sample size, we were unable to evaluate associations between body composition parameters and survival or adverse cardiovascular, pulmonary, or graft-related outcomes. The prognostic significance of the observed cross-sectional body composition patterns therefore cannot be determined from the present study. Larger multicenter prospective studies with repeated within-patient measurements, more comprehensive assessment of treatment, nutritional and rehabilitation-related factors, and complementary reference methods of body composition assessment are needed to confirm these findings and determine their clinical and prognostic significance.

## 5. Conclusions

In summary, heart and lung transplant recipients demonstrated distinct non-linear cross-sectional associations between body composition parameters and time since transplantation. Positive associations between absolute fat mass and time since transplantation were observed during the 3–9-month interval in both groups, whereas positive associations of SMM and FFM with time since transplantation were identified exclusively among LTx recipients.

## Figures and Tables

**Table 1:** Baseline characteristics and body composition of the study population.

Characteristic	Overall (*N* = 79)	HTx (*N* = 38)	LTx (*N* = 41)	*p* Value
Age (years)	49 (13)	47 (13)	51 (12)	0.13
Time since transplantation (months)	9 (3, 15)	9 (4, 14)	9 (3, 15)	0.5
Female	14/79 (18%)	4/38 (11%)	10/41 (24%)	0.11
Male	65/79 (82%)	34/38 (89%)	31/41 (76%)	
Height (m)	1.76 (0.09)	1.78 (0.09)	1.75 (0.09)	0.11
Relative fat mass (%)	29 (10)	32 (7)	26 (11)	0.002
Absolute fat mass (kg)	24 (12)	28 (10)	20 (11)	0.001
Fat-free mass (kg)	56 (11)	58 (10)	54 (11)	0.13
Skeletal muscle mass (SMM) (kg)	26 (6)	27 (6)	24 (7)	0.10
Phase angle (°)	4.34 (0.84)	4.36 (0.82)	4.33 (0.88)	0.9
ECW/TBW (%)	45.39 (2.79)	45.08 (2.38)	45.68 (3.13)	0.3

Data are presented as mean (SD), median (IQR), or *N* (%), as appropriate. ECW/TBW, extracellular water-to-total body water ratio.

**Table 2:** Detailed estimates of adjusted cross-sectional slopes. All estimates are adjusted cross-sectional slopes with 95% confidence intervals.

Variable	Group	Quartile	Estimated Cross-Sectional Slope per Month	*p*-Value
Relative fat mass	HTx	[1, 3]	0.40 (−0.23; 1.02)	0.212
		[3, 9]	0.36 (−0.12; 0.84)	0.143
		[9, 15]	0.24 (−0.16; 0.63)	0.239
		[15, 42.1]	−0.01 (−0.30; 0.28)	0.948
	LTx	[1, 3]	0.73 (−0.20; 1.66)	0.124
		[3, 9]	0.56 (−0.01; 1.13)	0.054
		[9, 15]	0.28 (−0.22; 0.78)	0.267
		[15, 42.1]	−0.48 (−0.89; −0.07)	0.021
Absolute fat mass	HTx	[1, 3]	0.80 (−0.19; 1.79)	0.114
		[3, 9]	0.72 (0.03; 1.42)	0.042
		[9, 15]	0.43 (−0.17; 1.03)	0.159
		[15, 42.1]	−0.14 (−0.53; 0.26)	0.497
	LTx	[1, 3]	1.04 (−0.12; 2.20)	0.080
		[3, 9]	0.83 (0.12; 1.53)	0.021
		[9, 15]	0.46 (−0.16; 1.08)	0.145
		[15, 42.1]	−0.49 (−1.00; 0.01)	0.053
Fat-free mass	HTx	[1, 3]	0.06 (−0.41; 0.53)	0.802
		[3, 9]	0.06 (−0.33; 0.44)	0.777
		[9, 15]	0.02 (−0.29; 0.33)	0.898
		[15, 42.1]	−0.15 (−0.40; 0.10)	0.235
	LTx	[1, 3]	0.61 (−0.15; 1.37)	0.116
		[3, 9]	0.52 (0.04; 1.00)	0.033
		[9, 15]	0.28 (−0.13; 0.69)	0.178
		[15, 42.1]	−0.24 (−0.59; 0.12)	0.193
Skeletal muscle mass	HTx	[1, 3]	0.18 (−0.21; 0.57)	0.359
		[3, 9]	0.17 (−0.11; 0.46)	0.231
		[9, 15]	0.12 (−0.12; 0.36)	0.339
		[15, 42.1]	−0.12 (−0.29; 0.04)	0.145
	LTx	[1, 3]	0.59 (0.03; 1.16)	0.039
		[3, 9]	0.49 (0.16; 0.81)	0.003
		[9, 15]	0.24 (−0.06; 0.53)	0.112
		[15, 42.1]	−0.14 (−0.36; 0.08)	0.205
Phase angle	HTx	[1, 3]	0.04 (−0.04; 0.12)	0.315
		[3, 9]	0.04 (−0.02; 0.10)	0.189
		[9, 15]	0.03 (−0.02; 0.08)	0.269
		[15, 42.1]	−0.02 (−0.05; 0.01)	0.194
	LTx	[1, 3]	0.06 (−0.01; 0.12)	0.081
		[3, 9]	0.05 (0.01; 0.10)	0.027
		[9, 15]	0.03 (−0.00; 0.07)	0.085
		[15, 42.1]	0.02 (−0.02; 0.05)	0.426
Extracellular water/total body water	HTx	[1, 3]	−0.13 (−0.34; 0.07)	0.202
		[3, 9]	−0.13 (−0.27; 0.02)	0.091
		[9, 15]	−0.09 (−0.22; 0.03)	0.145
		[15, 42.1]	0.03 (−0.05; 0.11)	0.449
	LTx	[1, 3]	−0.27 (−0.57; 0.02)	0.070
		[3, 9]	−0.24 (−0.41; −0.08)	0.004
		[9, 15]	−0.08 (−0.23; 0.07)	0.304
		[15, 42.1]	−0.06 (−0.17; 0.05)	0.276

## Data Availability

The raw data supporting the conclusions of this article will be made available by the authors on request.

## Abbreviations

The following abbreviations are used in this manuscript:

AFM	absolute fat mass
BIA	bioelectrical impedance analysis
BMI	body mass index
CAV	cardiac allograft vasculopathy
COPD	chronic obstructive pulmonary disease
ECW/TBW	extracellular water-to-total body water ratio
FFM	fat-free mass
FM	fat mass
HF	heart failure
HTx	heart transplantation
LTx	lung transplantation
PA	phase angle
RFM	relative fat mass
SMM	skeletal muscle mass
